# Case report: anaesthetic management of radical gastrectomy for gastric cancer associated with anti-N-methyl-D-aspartate receptor encephalitis

**DOI:** 10.1186/s12871-017-0379-2

**Published:** 2017-07-06

**Authors:** Lei Ding, Hongyu Tan, Ziyu Li, Jiafu Ji, Xuejun Song

**Affiliations:** 1 Key laboratory of Carcinogenesis and Translational Research (Ministry of Education/Beijing), Department of Anaesthesiology, Peking University Cancer Hospital & Institute, Beijing, 100142 China; 2 Key laboratory of Carcinogenesis and Translational Research (Ministry of Education/Beijing), Department of Gastrointestinal Surgery, Peking University Cancer Hospital & Institute, Beijing, 100142 China

**Keywords:** Case report, Anaesthetic management, Anti-N-methyl-D-aspartate receptor encephalitis, Gastric cancer

## Abstract

**Background:**

Anti-N-methyl-D-aspartate receptor (NMDAR) encephalitis is a rare neurological disorder that is caused by the production of antibodies against NMDARs. As many anaesthetic drugs interact with NMDARs and may worsen the disease and because the disease poses risks, such as cardiovascular events, hyperthermia and respiratory insufficiency, while under anaesthesia, administering anaesthesia to patients with this disorder is clinically challenging.

**Case presentation:**

A 55-year-old man with gastric cancer associated with anti-NMDAR encephalitis who was diagnosed 8 months prior was admitted to Peking University Cancer Hospital for tumour resection. Before surgery, the patient’s symptoms had been successfully controlled via aggressive immunotherapy. Radical gastrectomy was performed under general anaesthesia induced with remifentanil, propofol, and cisatracurium and maintained with sevoflurane and remifentanil. The patient had a favourable recovery without any adverse symptoms or post-operative complications.

**Conclusions:**

Adequate preparation for surgery is essential for the anaesthetic management of patients with anti-NMDAR encephalitis. These rare patients may benefit from general anaesthesia induced using remifentanil, propofol and cisatracurium and maintained using sevoflurane and remifentanil. Additionally, the use of NMDA antagonists, such as ketamine, nitrous oxide and tramadol, should be avoided.

## Background

Anti-N-methyl-D-aspartate receptor (NMDAR) encephalitis is recognized as a rare autoimmune-induced disorder in which autoantibodies generated in the body bind NMDA receptors in the brain, causing a series of neurological dysfunctions, such as psychosis, involuntary movements, autonomic nervous instability, central hypoventilation, seizure and hyperthermia [1–3]. Studies have demonstrated that these disorders commonly affect females in association with the presence of a mature NMDAR ovarian teratoma [2, 4, 5]. It has been reported that tumour resection in combination with aggressive immunotherapy (corticosteroids and/or immunoglobulin i.v.) can facilitate earlier functional recovery [6–8], but associated with a high rate of recurrence and mortality [9, 10]. Moreover, the interactions of various anaesthetic drugs with NMDARs may aggravate this disease and lead to increased risk with respect to general anaesthesia for tumour resection, such as cardiovascular events, hyperthermia and respiratory insufficiency; this risk poses a novel challenge for anaesthetic management during surgical procedures. This report provides the first description of anaesthetic management in a male patient with anti-NMDAR encephalitis who presented with gastric cancer. We discuss the specific considerations and certain principles related to the planning of general anaesthesia for the population of patients with anti-NMDAR encephalitis.

## Case presentation

The subject was a 55-year-old man (162 cm, 64 kg) with a 5-year history of stomach-ache that had worsened during the prior year. He also complained of hypologia, depression, insomnia and hypomnesis during the previous 2 years. Eight months prior to surgery at our hospital, he was admitted to Peking Union Medical College Hospital for medical evaluation immediately after he developed left upper limb twitching. Cerebrospinal fluid analysis revealed the following: cells 280 × 10^6^ L^−1^ (normal values 0–8 × 10^6^ L^−1^); lymphocyte 75% (normal values 49–73%); neutrophil 24% (normal values 0–2%); glucose 2.9 mmol·L^−1^ (normal values 2.8–4.5 mmol·L^−1^); protein 0.8 g·L^−1^ (normal values 0.1–0.4 g·L^−1^); IgG 61.30 mg·L^−1^ (normal values 10.00–40.00 mg·L^−1^); and positive antigen-specific oligoclonal bands and NMDAR antibody. Tests for paraneoplastic antibodies, including Hu, Yo, Ri, amphiphysin, CV2 and Ma2, were all negative. A previous PET scan and electronic gastroscopy did not show obvious lesions. The patient was diagnosed with anti-NMDAR encephalitis and treated with intravenous methylprednisolone and immunoglobulin for 8 days, followed by oral prednisone for 2 months. Eventually, his symptoms gradually improved. Two months later, gastroscopy and pathology revealed a malignant lesion in the gastric fundus and cardia (0.6 × 0.7 cm^2^). Based on previous reports, a neurologist suggested that the perioperative risk for this patient with anti-NMDAR encephalitis was extremely high if the patient underwent gastric cancer surgery, even if this surgery occurred during the recovery period, and recommended that the surgery be performed in our hospital—one of the most advanced and authoritative hospitals on gastric cancer surgery in China—by an experienced perioperative medical management team.

Radical gastrectomy was scheduled after the administration of chemotherapy for 4 months. Prior to surgery, the patient still exhibited a slight but very rare quiver in the left upper limb, but blood tests were all negative for NMDAR antibodies. Therefore, the patient was considered to be in convalescence. Dexamethasone (10 mg) was administered as a pre-anaesthetic medication. Upon the patient’s arrival to the operating room, invasive blood pressure monitoring was established. The patient’s blood pressure was 150/75 mmHg, his heart rate was 90 beats·min^−1^, and his arterial oxygen saturation was 97% when breathing room air. To ensure stable vital signs, vasopressors, beta blockers, anti-hypertensives and anti-cholinergics were readily available. General anaesthesia was intravenously induced with remifentanil (100 μg), cisatracurium (20 mg) and propofol (150 mg) to facilitate tracheal intubation and was maintained with oxygen (1 L·min^−1^), air (1 L·min^−1^), sevoflurane (1.5–2.0%) and remifentanil (0.3 μg·kg^−1^·min^−1^). Neuromuscular blockade was maintained with intermittent cisatracurium. The monitored parameters included ECG, ABP, capnography, pulse oximetry and the bispectral index (BIS). The patient’s intraoperative systolic blood pressure was 100–135 mmHg, his heart rate was 60–80 beats·min^−1^ and his BIS was 40–60. Surgery was completed without any complications. The durations of surgery and anaesthesia were 2 h 22 min and 3 h 18 min, respectively. Intraoperatively, total blood loss was 80 ml, urine output was 200 ml, and total infusion volume was 1600 ml. The patient was then transferred to the ICU with a tracheal tube and mechanical ventilation, administered a low dose of propofol for sedation and monitored closely. Considering that the patient was in convalescence from anti-NMDAR encephalitis, the neurologist suggested that there was no need to use corticosteroids or immunoglobulin. The patient was stable and successfully extubated two days after surgery. No evidence of post-operative complications or worsened neurological symptoms were observed. With an uneventful recovery and disappearance of the quiver, the patient was discharged on the 26th post-operative day after the risk of recurrence of anti-NMDAR encephalitis was evaluated. At the 2-month follow-up, he was symptom-free and exhibited no signs of any post-operative complications.

## Discussion

Anti-NMDAR encephalitis was first identified as a type of autoimmune limbic encephalitis in 2007. This disease has been commonly associated with malignancies such as germ cell tumours of ovarian teratomas and breast cancer but rarely gastric cancer, with unclear aetiology and incidence [2, 6–8, 11, 12]. Although there are several case reports on the anaesthetic management of anti-NMDAR encephalitis associated with ovarian teratomas, this report is the first description of the anaesthetic management of a patient with gastric cancer that led to this condition.

Anti-NMDAR encephalitis is classified as a paraneoplastic syndrome and involves the production of auto-antibodies against NMDARs induced by nerve tissue containing the NMDAR subunits in the tumour [2, 13]. Dysregulation of NMDARs has been linked to schizophrenia, Alzheimer’s disease and Parkinson’s disease [14, 15]. Inactivation of inhibitory gamma-amino butyric acid (GABA)-ergic interneurons, which express high concentrations of NMDARs, also plays a key role in the pathophysiology of this disease [13]. Clinical manifestations during the early stage are characterized by psychiatric symptoms. Late stages are often accompanied by paroxysmal sympathetic hyperactivity (PSH) that includes hyperthermia, tachycardia or hypertension, hypoventilation, and motor or complex seizures [13, 16, 17]. Various treatment modalities, including first-line immunotherapy (corticosteroids and/or IV Ig and/or plasma exchange) and the early removal of an underlying tumour, may be associated with a good prognosis. However, recovery may require 3–4 months of hospitalization followed by several months of rehabilitation. Moreover, 12% of patients relapse [9, 10].

Patients undergoing surgical resection of tumours commonly require general anaesthesia. However, anti-NMDAR encephalitis presents anaesthesiologists with many risks and challenges.

First, many anaesthetic drugs inhibit NMDAR and can therefore induce the same symptoms as those observed in anti-NMDAR encephalitis [13, 18]. N_2_O reduces NMDAR-mediated excitatory currents in the basolateral amygdala, an area associated with anaesthesia-induced amnesia and the formation of aversive memories, fear, and addictive behaviour [19]. Ketamine is a well-known NMDAR antagonist that inhibits glutamate-triggered calcium influx [20]. The effects of certain anaesthetic drugs on NMDARs remain unclear. Research has demonstrated that halogenated anaesthetics, such as sevoflurane, inhibit NMDA-gated currents and NMDA-induced mitochondrial membrane depolarization; however, their enhancing effects on GABA receptors may be dominant and may blunt the autonomic hyperactivity observed in patients with anti-NMDAR encephalitis [1, 21–25]. Propofol acts by enhancing GABAergic transmission and may also inhibit NMDARs in vitro [4, 26, 27]; however, the clinical relevance of this inhibition has not been established. Moreover, drugs that primarily act on GABA receptors, including pentobarbital, diazepam, and midazolam, may also have indirect interactions with NMDARs. Sufentanil and cisatracurium appear to have no significant effects on NMDARs [28].

Therefore, when administering anaesthesia to patients with anti-NMDAR encephalitis, it is wise to avoid the use of drugs that act via NMDARs, including ketamine, N_2_O and tramadol. Medications that indirectly interact with NMDARs can still be considered for use. Several important medications, including fentanyl, sufentanil, remifentanil, propofol, sevoflurane, isoflurane, desflurane, vecuronium, rocuronium and cisatracurium, have been well tolerated during surgery in certain reported cases. Chen W et al. [29] reported three cases that underwent anaesthesia for ovarian teratoma resection with midazolam, fentanyl, propofol and rocuronium for induction and sevoflurane, fentanyl and rocuronium for maintenance. All patients survived the surgery and were discharged with mild psychiatric symptoms. Lapebie et al. [30] hypothesized that anaesthesia using both propofol and sevoflurane simultaneously may facilitate inhibition of the NMDA pathway and worsen the clinical presentation of anti-NMDAR encephalitis. However, in most cases, a detailed anaesthetic procedure was not provided, and the post-operative course was not described. Therefore, the choice of anaesthetics for patients with anti-NMDAR encephalitis requires additional study.

Furthermore, due to the complex condition of patients with anti-NMDAR encephalitis, including high fever, autonomic dysfunction, and central ventilation dysfunction, attention should be paid to adverse reactions, such as cardiovascular events, hyperthermia, respiratory insufficiency and delayed (or difficult) extubation, during the induction and maintenance of general anaesthesia and after surgery. Anaesthesiologists should also be prepared for PSH, which is frequently observed in combination with this disorder. The readily available vasopressors, beta-blockers, anti-hypertensives, and anti-cholinergics during a case are prudent to ensure that any autonomic instability can be dealt with in a timely manner. Because PSH has been described in patients suffering from severe traumatic brain injury, the management principles for PSH should be followed for anti-NMDAR encephalitis patients [31, 32].

The conditions of cases reported by Chen W et al. [29] and Lapedbie et al. [30] all deteriorated to some degree after anaesthesia, although all cases ultimately survived. In another hospital, 3 patients with similar conditions who recovered after immunotherapy unfortunately relapsed several days after anaesthesia for ovarian teratoma resection and did not survive to discharge (not reported). This outcome suggests that certain forms of anaesthesia may play significant roles in inducing recurrence and death among patients with anti-NMDAR encephalitis.

In our patient, we used remifentanil, cisatracurium and propofol to induce anaesthesia and sevoflurane, remifentanil and cisatracurium for maintenance. Vasopressors, beta-blockers, anti-hypertensives and anti-cholinergics were readily available. As Lapebie et al. [30] reported a case who was administered sevoflurane and propofol during the operation and a high dose of propofol for post-surgery sedation. We hypothesized that his deterioration after surgery may have been attributed not only to the combination of sevoflurane and propofol but also to the high dose of propofol. Thus, we used a low dose of propofol only during induction and chose sevoflurane as the single anaesthetic during maintenance. All medications were given at the minimum effective dose and stopped as soon as the operation was completed to reduce potential reactions with NMDARs. As mentioned above, patients with anti-NMDAR encephalitis may also exhibit autonomic dysfunction and central ventilation dysfunction, and some anaesthetic drugs may induce or aggravate cardiovascular events, respiratory insufficiency and delayed (or difficult) extubation. Several cases deteriorated after extended periods of time following surgery. Early extubation without adequate time of observation, assessment or medication in some cases may induce or worsen cardiovascular events or result in reintubation if the patient relapsed. To ensure adequate time to nurse and monitor the patient and to provide safe and stable post-operative recovery, we kept the patient intubated and sedated with a low dose of propofol, as propofol is a short-acting drug that may not affect NMDARs sufficiently to worsen neurologic symptoms. The patient was extubated after observation for one day, and we ensured there was no sign of deterioration or dysfunction of spontaneous breathing. In the report by Chen W et al. [29], the three patients were extubated on the 1st, 5th and 90th day and discharged on the 3rd, 5th and 8th week, respectively, after surgery, depending on the status of anti-NMDAR encephalitis. However, all patients developed obvious neurological and psychiatric symptoms and received immunoglobulin after surgery. By contrast, our patient was extubated and discharged early and recovered well without any neurologic symptoms, recurrence or worsening post-surgery, even in the absence of immunotherapy after surgery. We suspect that this result may be attributed to the reasonable management of anaesthesia (without midazolam) and the effective control of his disease prior to surgery.

## Conclusions

In this case report, we present successful anaesthetic management of radical gastrectomy for gastric cancer associated with anti-NMDAR encephalitis and highlight specific aspects of anaesthetic management for this rare type of encephalitis. For patients with this encephalitis, NMDA antagonists, such as ketamine, nitrous oxide and tramadol, should be avoided, whereas benzodiazepines, opioids, muscle relaxants and curares, which have been demonstrated to have no significant effects on the NMDA pathway, are preferred. Medications that have indirect interactions with NMDARs can be considered but should be used with caution. Finally, adequate preparation, including ensuring the availability of vasoactive agents and monitoring throughout the perioperative period, particularly after surgery, is of vital importance.

There is scarce literature to date describing anaesthetic management for patients with anti-NMDAR encephalitis, and further investigations are necessary to develop detailed guidelines for anaesthesiologists to assess surgery-related risk factors of anti-NMDAR encephalitis and to develop a stable perioperative anaesthetic plan for patients with this condition.

## Abbreviations

BIS: Bispectral index
GABA: Gamma amino acid butyric acid
NMDAR: N-methyl-D-aspartate receptor
PSH: Paroxysmal sympathetic hyperactivity
